# The Pathology of Alveolar Abscess

**Published:** 1888-03

**Authors:** E. Lloyd Williams

**Affiliations:** Assistant Dental Surgeon, London Dental Hospital.


AMERICAN JOURNAL

—OF—

DENTAL SCIENCE.

VOL. XXI. THIRD SERIES—MARCH, 1888. NO. 11.

ARTICLE I.

### THE PATHOLOGY OF ALVEOLAR ABSCESS.

BY E. LLOYD WILLIAMS, M. R. C. S., L. R. C. P., L. D. S. ENG.,

(Assistant Dental Surgeon, London Dental Hospital.)

### DEFINITION.

The term alveolar abscess has been used so loosely amongst dental surgeons, and its pathology so variously understood—at least, if we can gauge the latter point from observations of common practice—that it would be extremely hazardous to analyse the various opinions entertained, and attempt to strike a mean which might fairly be said to represent the average opinion. Under these circumstances, it becomes difficult to attempt a definition within limits at once exact and comprehensive. I think, however, that I shall not trespass far afield in submitting, that "alveolar abscess is a circumscribed collection of purulent materlal as one of the results of inflammation of the alveolar-dental periosteum commencing around the root or roots of an individual tooth." This definition may appear at first to be somewhat arbitrary, if not sufficient; but it has at least the merit of cutting off a variety of suppurative affections, ranging from mercurial periostitis to Riggs' disease, and should prove acceptable if only on the ground that it thus narrows down the subject within the practical limits of a short paper.

### ETIOLOGY.

In the vast majority of cases, the cause may be distinctly traced as a sequence of the death of a pulp, and a direct spread of inflammation from the point where the vessels at the apex of the root of tooth are in immediate relation with the dental periosteum. And, inasmuch as the death of a pulp usually occurs after exposure to the external air, it is impossible in such cases to exclude the septic origin of the initial periostitis. But abscess may ensue on the death of a pulp after traumatic injury without access of air, and here the causation becomes more difficult to determine. And there is yet another form—idiopathic abscess—which is far from uncommon, where there has been neither exposure of pulp or traumatic injury, and the tooth itself is apparently sound and normal in colour. This latter condition, which is probably due to nutritive changes which are difficult to trace, was well illustrated in a mouth which I once examined, where a lady about twenty years of age had three typical sinuses apparently in connection with the sockets of three lower incisor teeth to all appearance sound and healthy. Further interest was attached to the case from the fact that the patient stated that she suffered from a similar state of the mouth for several years during the spring. The condition passed away in a short while without active interference, and did not recur up to the time I lost sight of the case.

In a tooth where pulp has died as the result of a blow, without access of air, the pulp itself may or may not be found to be in a state of putrefactive decomposition. My own experience leads me to believe that in the majority of such cases a septic condition exists, and on this point I am compelled to differ from Mr. Charles Tomes, who brought this interesting subject before the Society some little while back. It is by no means uncommon for suppurative collections in different parts of the body to be found in a stinking condition, an analogous state of things, which is worthy of passing note, being found in cases of abscess in bone where the pus is foul and greenish in colour. The precise relation of organisms to the processes of putrefaction and fermentation is a subject which excites much variety of opinion amongst modern pathologists. Believers in Pasteur's germ theory think that these processes are induced by organisms; whilst those who object to this theory contend that putrefaction and fermentation may be initiated without the presence of bacteria, and that the organisms themselves are generated from the organic constituents dissolved in fermentable fluids. If the latter theory should prove correct, then the occurrence of putrefaction in a pulp which has broken down without being exposed to the air is but a normal pathological condition. On the other hand, if the germ theory be true, it is not difficult to believe that tissue whose vitality has become lowered, or altogether destroyed, may cease to resist the putrefactive influence of germs which, it is quite possible, may exist in normal blood, even though they be difficult of demonstration. Experiments have been made on animals by feeding them on phosphorus and thus lowering their vitality, and in these cases micrococci have been distinctly traced in the blood. That the blood under certain conditions is capable of circulating infective material is now generally accepted, as instanced in *septicæmia* and *pyæmia* and that the blood may harbour hæmatozoa has been demonstrated beyond cavil in the peculiar tropical disease known as *chyluria*.

There is another point with regard to the possibility of infection from another source, which is suggested by a statement made at the last annual meeting of the British Dental Association by the President, Mr. Brownlie, who unhesitatingly affirmed that a tooth is not in itself water-tight. I cannot personally endorse that statement; on the contrary, I believe that to be entirely erroneeus, although I have not yet had the opportunity of carrying out a sufficient number of experiments to disprove the suggestion. If, however, a tooth in its normal condition does leak, another source of infection from the fluids of the mouth becomes easily conceivable.

### VARIETIES AND CLINICAL CHARACTERS.

The principal varieties of alveolar abscess may be characterised as (1) *acute* and (2) *chronic*.

(1.) The term *acute* as applied to an abscess of any description, is indicative of an intense form of inflammation, generally dependent upon any injury whose action is short in duration and severe in character. Perhaps the commonest cause of acute alveolar abscess is a sharp attack of inflammation followed by its death. Clinically, it is characterised by an intense inflammation of the alveolo-dental membrane whicn extends to the adjacent alveolus and gum, and may largely infiltrate the overlying and continuous structures. The inflammatory process seems to focus itself somewhere near the point of initial injury, and the abscess generally bursts on the surface of the gum in the immediate neighbourhood of the offending tooth. In a favourable case, when the pus is evacuated either by nature or the surgeon, the inflammatory action generally subsides gradually, and resolution of the remaining products appears to take place. In a less favourable case the acute passes into the chronic stage.

(2.) *Chronic* alveolar abscess is much less intense in character, is often show in asserting itself, and is generally consequent on the death of a pulp. It may develop with little or no pain, and often only proclaims its presence in consequence of treatment of a pulpless tooth. A limited area of inflamed tissue within the socket ultimately breaks down, and the discharge which may vary in character, seeks the surface by way of an empty root canal or by a sinus through the overlying alveolar plate, opening by a papilla-like orifice on the gum. More rarely the pus may find vent by passing between a detached periosteum and the tooth, thus gaining the surface at the margin of the gum. An abscess of this description may go on for an indefinite time, and seems to be capable of taking on alternate periods of activity and quiescence. Chronic abscesses in other parts of the body occasionally cease to discharge when the irritation causing them terminates, and they may diminish by absorption of their fluid components—whilst the solid elements become dried up and shrunken into a putty-like mass. They may remain in this state without giving rise to any trouble, or they may again become the seat of suppuration, and form what Sir James Paget has termed "residual abscess." A similar state of things in a modified form may happen in the mouth, and it is far from uncommon to note instances, which, I think, may be appropriately called "residual alveolar abscess."

The area of inflammation is generally limited, but the ultimate sequlæ may be serious, involving necrosis of bone and destruction of the soft tissues; whilst the pus may eventually burrow through a track which often opens at a great distance from the socket of the errant tooth. As an instance of destructive inflammation of this kind, the specimen handed round is a good example, in which necrosis of the superior maxilla extended from the upper canine to the wisdom tooth on the same side involving the floor of the antrum, as the result of a chronic alveolar abscess in connection with the fang of the first bicuspid tooth. As an instance of the burrowing of pus from a chronic abscess, of which there was absolutely no evidence in the mouth, I may mention a case in my own practice where a canal stood out in contour on the neck of the patient about the size of a goose quill, and eventually bifurcated, each branch ending in a pouch about an inch above the clavicle, which, when full, discharged itself by ulceration of the skin.

But however interesting it might be to discuss fully the clinical aspects of the subject, it is not within the scope of the present paper; but rather to confine your further consideration to the purely pathological phenomena of ordinary cases. And, to this end, I would ask your attention exclusively to the changes which occur in the various tissues involved.

First of all, we may consider the alveolo-dental periosteum, which plays a prominent part; and, inasmuch as the changes which take place may be studied with greater facility in chronic affections, the latter type of inflammation will be taken as illustrating the various processes which I propose to examine. The membrane itself is, as you know, composed of connective tissue of a moderately dense character, and the ordinary primary stages of inflammation common to this type of tissue are in no way different from those which occur in the alveolo-dental membrane. We will, therefore, pre-suppose the infiltration of the tissue with leucocytes. This inflammatory process may end in (1) resolution, (2) organization, or (3) suppuration.

1. *Resolution.*—There is no reason to suppose that this process is uncommon in the alveolo-dental membrane, although it would, of course, be difficult to produce histological specimens—for obvious reasons. We are clinically familiar with cases of threatened abscess which disappear rapidly, where, no doubt, the primary stages of hyperæmia and infiltration do occur, only to be followed by the fortunate removal of the inflammatory products.

2. *Organization.*—Failing resolution, the new cells with which the tissue is infiltrated become organized into a new tissue. This "productive" action is common in chronic inflammation, and is dependent on a low degree of inflammatory action. The process of organization is demonstrable to the naked eye by the palpable thickening of a membrane which often retains its attachment to a tooth when extracted, and it is under these conditions that we are enabled to study the changes that occur. On making a section of a thickened periosteum, there are one or two things which enforce their identify upon us before making a minute examination. The first is, that the original oblique direction of the fibres is lost, and in its place the new fibrous tissues lies parallel with the root and its socket. The second is, that the intensity of the inflammatory action has been confined to the lower third (or apical portion of the membrane.) And the third is, that although the membrane may be variously affected in various degrees, yet no part entirely escapes the inflammatory action. The new tissue, if examined more minutely, will be found to be of two principal varieties. That which is more highly developed is fibroid in character; it is dense in structure, and consists of closely-packed wavy fibres in which spindle-shaped elements figure conspicuously. A less highly-developed structure consists of a loose meshwork of adenoid character. In the early stage, some of the larger cells range themselves round, and enclose groups of smaller cells; later on, the walls become condensed and fibrillated. This latter tissue is constantly present in low inflammatory conditions, and is often associated with teeth which are the subjects of periosteal irritation whilst their pulps are still in a healthy condition.

With regard to the degeneration which may occur, one would naturally look for a fatty change; but although this is quite possible, I have been unable to trace a single instance of fatty degeneration. Caseous degeneration is not common, but I have come across evidences of it in a few specimens.

3. *Suppuration.*—When the inflammation is intense or prolonged, the leucocytes accumulate in sufficient numbers to form pus, at the expenses of entirely destroying the tissue at that spot. This is what has already been referred to as the "breaking down" of tissue. The old idea of an alveolar abscess—and one still tenderly nursed—was that a pyogenic membrane was formed which had a special function of secreting pus, the latter denuding the fang extensively. Even now the enlarged periosteum often clinging to an extracted root is looked upon as the wall of an "abscess sac," and this wall is mysteriously connected with any purulent discharge in the neighbourhood. We all know how a chronic alveolar abscess may take on active inflammatory action with the result of a large accumulation of pus, but, as a rule, the action is of a low type, and the area of tissue broken down is comparatively small. I am compelled to disagree with the usual account given in text books about the denudation of fangs which are supposed to be bathed in pus as a regular thing. In extreme cases where the hard tissues are undoubtedly necrosed (in the trve sense of the word) the fangs will of course be in actual contact and be largely denuded; but that this is a common factor in ordinary cases I deny altogether. Pus is supposed to be endowed with the power of absorbing the tissues with which it comes in contact, and by virtue of this power it has been considered the chief factor in the absorption of roots. I believe, however, that the latter is due to another agency altogether, which will be considered presently. The nature of the discharge from an alveolar abscess varies in character from what is known as laudable "pus" to a thin sanious exudation. It has a strange phosphatic odour peculiar to itsrlf, which cannot be accounted for, as far as I am aware, any more than the peculiar odour of sweat in acute rheumatism; like it, however, it has its own diagnostic value. There is one point in connection with alveolar suppuration which becomes important clinically, and that is, that no pronounced inflammatory discharge from a periosteum can be detected, except the pulp in connection with it be completely broken down. A local suppurative periostitis, such as I have described, may be altered in character and develop into a serous cyst with a thin papery bony wall; and in several of these cases of maxillary cyst I have been enabled to distinctly trace the history of alveolar abscess.

CHANGES IN CEMENTUM.

The phenomena of absorption and addition to the surface of cementum are present in a marked degree in connection with chronic alveolar abscess. The latter condition has been described as exostosis, hyperostosis, and hypertrophied cementum. The last term is incorrect, for hypertrophy only occurs in connection with the increased functional activity of a part; the two first are misleading. In lieu of something better, I would suggest the term "cementosis," which is more expressive of what actually takes place. Absorption and cementosis may go on separately, synchronously, or alternately in the same root; and it seems quite impossible to decide upon the exact factors which determine and govern the various processes. As a broad general rule, I think it may be stated that a slight degree of irritation may produce cementosis, whilst absorption will depend upon more active inflammatory action.

1. *Absorption.*—As already stated, absorption of a root may proceed in a most erratic fashion, and inasmuch as a deposit of cementum may be taking place at the same time in the same specimen, it becomes somewhat difficult to trace the process. It is an easy matter to examine an inflamed periosteum in a comparatively fresh condition, but, unless the corresponding surface of the tooth be also observed at the same time under the microscope, we are apt to imagine a local condition which may not possibly exist. The various diagrammatic drawings which we see scattered about in popular works are only too often testimonies to vivid imagination rather than to truthful observation. If we want to find out what really does take place in the pathological absorption of the root, a section must be cut of both root and membrane, so that their exact relationship may be observed. This entails prolonged manipulation, which cannot but be prejudicial to the soft tissues; hence the difficulty by which we are handicapped. Nevertheless, a good deal may be grasped, and the details supplied may be supplemented by fair inference. There is one fact that obtrudes itself upon anyone who has studied the microscopic appearances of absorption of teeth, and is,—that pus has little or nothing to do with the immediate process. The surface of the hard tissue presents numerous absorption facets, forming on section segments of irregular circles, and in favourable specimens the individual facets are seen to certain large protoplasmic masses with a great number of neucle; these gaint cells are backed up by cells similar in character but smaller in size, whilst beyond they imperceptibly graduate into ordinary leucocytes, supported by a fibrous stroma; and the direction of the fibres is worthy of note, as being in the direction of the absorption cells, and often at right angles to the fibrous tissue surrounding the root. That these cells are recruited from the leucocytes there can be no doubt, but what the nature of their solvent action is, remains at present a riddle.

2. *Cementosis* is the result of a low inflammation, as instanced in the case of teeth which cannot be detected as unhealthy in the mouth, whilst absorption is undoubtedly the result of a higher inflammatory action. The processes are, therefore, dependent upon local nutritive conditions of the alveolo-dental membrane, and variations of these conditions must account for the variation of the processes. The varieties of cementum deposited may be grouped into four divisions:— (1) Granular, (2) Laminar, (3) Lacunar, (4) Irregular. All of these varieties may be found in cementosis as the result of chronic thickening of the periosteum in a single specimen. In the normal cementum covering ordinary teeth, where the tissue is thin, it will be found faintly granular and almost structureless, whilst in positions where it is thick we have lacunæ and canaliculi pretty regularly distributed. The cementum found in conical molars where the fangs are glued together will generally be found to be of the granular variety. The laminer variety stains deeply, and looks as if wavy fibres running parallel with the surface of the root had become calcified; it is common in cementum covering the roots of teeth which have been slightly inflamed—as in the case of teeth with healthy pulps. Lacunar cementum is not found in large quantities on roots which have been the subject of tolerable intense inflammation, but occurs in isolated patches, and the lacunæ and canaliculi are irregularly distributed. The irregular variety, on the contrary, does appear in these cases, and suggests the calcification of a jumbled mass. Both this variety and the granular have faint linear markings. I am unable to find out what variety of circumstances regulate the type of tissue formed, or to discover how the cell-elements differ either in size or arrangement in producing the different varieties. It has been suggested that the genetic cells (osteoblasts) are in no way to be distinguished from absorption cells (osteoclasts,) but I think they may be distinctly differentiated. In the first place, they are distinctly smaller than the giant absorption cells; and in the second place, they are supported by a tissue whose fibres generally run parallel with the surface of the root. Should, however, the fibres happen to deviate from this general direction, the linear marking will correspond with such deviation.

### CHANGES IN THE ALVEOLUS.

The bone lying in immediate contact with an inflamed dental periosteum is liable to become absorbed, and a specialised tissue performs this process in a precisely similar manner to that which obtains in the absorption of cementum. The space thus gained is filled with granulation tissue, and the inflammation may eventually cut off the vascular supply of a portion of bone, which then becomes necrosed. The medullary tissue, with which the alveolus may become riddled, cannot be distinguished in any way from the continuous tissue surrounding an inflamed root.

### CHANGES IN DENTINE.

There has been, and there still exists, a great conflict of opinion as to whether "vital action" occurs in dentine, and whether strictly pathological condtitions may be observed in this tissue apart from any changes which can be accounted for by purely chemical action. The question has arrived at its acutest stage in discussing the phenomena of caries, but as caries and alveolar abscess have no intimate relation the one to the other, I intend giving the former a wide berth. It will at least be a slight novelty—if of less interest—to enquire if any changes occur in dentine as the result of periostitis; and if so, whether they may be considered as pathological. It has been commonly noted that the dentine of the roots of teeth in old people has a peculiar horny, translucent appearance, quite distinct from the ordinary characteristic opacity of ordinary teeth; and this is also true of the roots of teeth exposed to long periosteal mischief. If the latter be carefully examined in section, there appears to the naked eye a distinct outer belt of tissue more apparent than the rest of the dentine, and less susceptible to staining. The microscopic appearances confirm the existence of this transparent belt, and one naturally seeks for an explanation. In trying to account for the "transparent zone" in connection with caries, several theories have been advanced to explain a condition which appears to be allied to that under consideration. The simplest way of explaining the increased transparency is to ascribe it to calclfication of the dentinal fibrils, and this is the ground taken up by Dr. Magitot. Leber and Rottenstein take up the direct negative, and say that the transparency is due to decalcification. Professor Wedl seems inclined to deny the theory of calcification, and says he has succeeded in staining the tubes and their contents of the transparent teeth of old people with carmine. Other workers have experimented in this direction, but time will not permit of an analysis of their opinions. Mr. Charles Tomes, to whom we are indebted for so much research in the field of dental pathology, appears to disfavour the idea of vital action in dentine—at least, that is the impression conveyed by a careful perusal of his latest account of caries. I am afraid my own experiments are so slight as scarcely to justify the expression of an opinion, and yet I am unable to account for the transparent belt of dentine in fangs which have been involved in periosteal inflammation without assuming a process of a low inflammatory nature. In the first place, it has yet to be proved that the tissue is incapable of pathological changes *per se*; the fact that it is non-vascular is of itself not sufficient, and on *a priori* grounds we are justified in assuming that any tissue, if it be alive, is capable of exhibiting inflammatory action. From a large number of sections which I have made, there appears to be a distinct increase of medullary tissue in the shape of enlarged tubes and their contents, which is more marked as it recedes from the area of irritation in the direction of the pulp canal. And this is further explained by its liability to deep staining, a phenomenon which is a characteristic feature of inflammatory material. On the other hand, the transparent belt already referred to in immediate continuity with the cementum certainly gives evidence of calcification as a sequence of inflammatory action; it may be noted that the increased transparency of the tissue may be differentiated from the dentine nearer the pulp canal even after the tooth has been softened in acid for the purpose of section cutting. In fact, the process seems to be somewhat analagous to sclerosis of bone, varying in degree rather than in type. It has been generally assumed, in the words of Mr. Charles Tomes, that the vitality of the dentine is sacrificed when the pulp is destroyed; and we have been in the habit of speaking of a pulpless tooth as a *dead tooth.* I cannot subrcribe to this formula, because I believe that death of the dentine does not necessarily follow death of the pulp. I think I may assert, without fear of contradition, that in the whole range of human pathology not one instance could be found of new living material being deposited on dead tissue. Yet this is true if death of the dentine follows death of the pulp, as the deposition of cementum to repair absorbed patches of dentine in pulpless teeth may be easily demonstrated. One of the specimens under the microscope clearly shows this, and is taken from one of the fangs of a lower molar which had been treated for alveolar abscess unsuccessfully for a long period. This last indication of vital action in dentine is one which appears to me to be convincing; and I would particularly invite those members of the Society who are skeptical of anything in the shape of a pathological condition of dentine—and they must be in a large majority—to discuss this particular point, and try and account for such a strange phenomenon.—*Dental Record.*

